# A high-density genetic linkage map and QTL mapping for growth and sex of yellow drum (*Nibea albiflora*)

**DOI:** 10.1038/s41598-018-35583-1

**Published:** 2018-11-22

**Authors:** Changliang Qiu, Zhaofang Han, Wanbo Li, Kun Ye, Yangjie Xie, Zhiyong Wang

**Affiliations:** 1 Key Laboratory of Healthy Mariculture for the East China Sea, Ministry of Agriculture; Fisheries College, Jimei University, Yindou Road, Xiamen, 361021 P.R. China; 2 Laboratory for Marine Fisheries Science and Food Production Processes, Qingdao National Laboratory for Marine Science and Technology, Qingdao, 266235 P.R. China

## Abstract

A high-density genetic linkage map is essential for the studies of comparative genomics and gene mapping, and can facilitate assembly of reference genome. Herein, we constructed a high-density genetic linkage map with 8,094 SNPs selected from 113 sequenced fish of a F1 family. Ultimately, the consensus map spanned 3818.24 cM and covered nearly the whole genome (99.4%) with a resolution of 0.47 cM. 1,457 scaffolds spanning 435.15 Mb were anchored onto 24 linkage groups, accounting for 80.7% of the draft genome assembly of the yellow drum. Comparative genomic analyses with medaka and zebrafish genomes showed superb chromosome-scale synteny between yellow drum and medaka. QTL mapping and association analysis congruously revealed 22 QTLs for growth-related traits and 13 QTLs for sex dimorphism. Some important candidate genes such as *PLA2G4A*, *BRINP3* and *P2RY1* were identified from these growth-related QTL regions. A gene family including *DMRT1*, *DMRT2* and *DMRT3* was identified from these sex-related QTL regions on the linkage group LG9. We demonstrate that this linkage map can facilitate the ongoing marker-assisted selection and genomic and genetic studies for yellow drum.

## Introduction

A genetic linkage map with high density and resolution is a critical and indispensable tool in a wide range of genetic and genomic researches^1^. The density and resolution of a linkage map mainly determined by two factors: the number of mapping individuals, and the type of candidate mapping markers and the corresponding accuracy of genotyping^2^. In general, the more mapping individuals in the mapping family, the higher density and resolution of a linkage map can be obtained. Nevertheless, sequencing more mapping individuals means the increase of sequencing costs. Thus, the significant improvement of linkage map resolution is commonly accompanied by the technical revolution of genetic marker development^2^. For many important aquatic species, such as channel catfish^3^, Pacific oyster^4^, tilapia^5^ and white shrimp^6^, most of their first generation linkage maps were constructed using both dominant and codominant markers, such as AFLP (amplified fragment length polymorphism) and SSR (simple sequence repeat) markers^2^. However, these maps demonstrate a limited capability in the application of fine mapping of QTLs or marker-assisted selection (MAS) due to their low density and resolution which mostly caused by the intrinsic limitations of mapping markers^2^. Although SSR markers have the advantages of low cost of genotyping and high level of polymorphisms, the lab work to construct a linkage map with SSR markers is laboursome and time-consuming^7^. Recent advances in the next-generation sequencing technologies (NGS) have enabled rapid and cost-effective discovery and genotyping for genetic markers, mainly in the form of single nucleotide polymorphisms (SNP), which greatly promote the construction of high-density SNP-based linkage maps^7^. Now, high-density SNP-based linkage maps have been constructed for a series of economically important fish species, such as large yellow croaker^8^, bighead carp^9^, common carp^10^, Atlantic salmon^11^, channel catfish^12^, Japanese flounder^13^.

A high-density genetic linkage map is one of the essential prerequisites for fine mapping of quantitative trait loci (QTL), positioning of candidate genes, marker-assisted selection, comparative analysis of genomic synteny and assembly of chromosome-level reference genome^2^. In the past decades, linkage maps for a series of aquatic species have been constructed using different genetic markers, such as catfish^12,14^, carps^15–17^, tilapia^5,18^, Asian seabass^7,19^, Japanese flounder^13,20^, Atlantic salmon^11,21^, rainbow trout^22,23^. High-density linkage maps act as chromosomal framework for QTL mapping, and facilitate marker-assisted selection in many aquatic species. For instance, QTLs underlying growth-related traits had been localized in many fish species, such as Asian seabass^24^, rainbow trout^25^, salmons^26^; sex-controlling loci have been mapped in the assistance of linkage map in halibut^27^, tilapia^28^, and half-smooth tongue sole^29^; lymphocystis disease-resistance locus in Japanese flounder has been successfully located and widely used in marker-assisted breeding^30^; Infectious Pancreatic Necrosis resistance loci in Atlantic salmon have been localized and utilized in germplasm screening^31^. Moreover, linkage maps were particularly preferred in comparative genomic analysis of studying chromosomal fragment rearrangement and evolution^11^. For instance, comparison of the croaker genome with other five teleost species revealed that many teleost underwent inter-chromosomal rearrangements after speciation from a common ancestor^8^; in addition, BLAST search of the reference sequences with SNP markers in the linkage map of nine-spined stickleback against the three-spined stickleback genome sequences indicated a high degree of genomic synteny^32^. Furthermore, high-density linkage maps can act as the chromosomal framework for genome sequences assembly, and even assist the assembly of chromosome-scale reference genome. For instance, with the aid of a linkage map of catfish, 1,602 scaffolds of the reference genome were anchored to 29 linkage groups, covering over 97% of the total genome assembly^33^; what’s more, a linkage map constructed for genome assembly validation in oyster indicated widespread errors on whole genome assembly^34^.

Yellow drum (*Nibea albiflora*), belonging to the family of Sciaenidae, is a commercially important perciform fish species in East Asia^35^. The mariculture of this species dates back to the 1990s and has rapidly spread throughout the coast of southeast China in recent years^36^. The great expansion of large-scale yellow drum farming has led to an increasing demand to promote aquaculture traits using MAS. In the past two decades, studies have been carried out in the areas of embryonic development, pre-larva morphology and seed production^37,38^. Nevertheless, very limited genetic and genomic resources have been documented, including a mitochondrial genome^39^, hundreds of AFLP^40^, SSR markers^41–43^ and expression sequence tags (ESTs)^44^, which severely impeded the genetic improvement and breeding programs of this species. Recently, the genome of yellow drum has been sequenced in our group, laying the foundation for the genetic and genomic studies of this species (unpublished data). Given the significance of linkage map in genetic and genomic researches, we set out to construct a genetic linkage map for the yellow drum with a sequenced large F1 family. In this study, we reported the construction of a high-density genetic linkage map with 8,094 SNP markers, which was the first version of linkage map for yellow drum. Comparative analysis of genomic synteny with medaka and zebrafish genomes gave us new insights of yellow drum genome evolution. Subsequently, QTL loci were located and candidate genes in the corresponding QTL regions were identified for growth and sex.

## Results

### Genome resequencing and genome-wide SNP discovery

The genome size of the yellow drum was previously estimated to be ~620 Mb^45^. Genome resequencing was performed for 2 parents and 111 progenies with 150-bp pair-end sequencing strategy on Illumina HiSeq X10 platform. 19.5 Gb and 20.4 Gb clean data was obtained for the female and male parent, equivalent to 31.5× and 33.0× genome coverage, respectively. For 111 offspring, on average of 3.9 Gb sequencing data was obtained, equivalent to 6.3× genome coverage.

The SNPs were called using GATK UnifiedGenotyper method^46^ (see Methods). In total, 4.3 million raw SNPs were obtained. After filtration with stringent criterion (minor allele frequency (MAF) >5% and SNP calling rate >95%), approximately 1.2 million were retained for further analysis, including 185,922 and 196,819 SNPs heterozygous only in female and male parent, respectively. We further selected 153,722 female markers and 158,825 male markers after filtering of non-Mendelian inheritance sites (P < 0.01).

### Linkage mapping

The marker number used for linkage mapping was further filtered because of the poor performance of JoinMap 4.0 in computing large markers set. For each 4 Kb region with multiple markers, only the marker with the highest sequencing coverage was retained. Sex-specific maps were primarily constructed using these SNP markers which were heterozygous only in female (AB♀ × AA♂ or AB♀ × BB♂, 4688 SNPs) or male parent (AA♀ × AB♂ or BB♀ × AB♂, 4588 SNPs), respectively. Subsequently, the sex-specific maps were further integrated using 562 anchor markers which were heterozygous in both parents (AB♀ × AB♂). In total, 5,250 and 5,150 SNP markers were used for the construction of female and male linkage map, respectively.

For both sex-specific maps, 24 linkage groups were obtained, corresponding to the haploid chromosome number of *N*. *albiflora*^47^. In detail, the female map contained 4,361 SNP markers and spanned 2660.82 cM with an average marker interval of 0.61 cM (Supplementary Fig. S1). The genetic length of each LG ranged from 52.5 cM (LG11) to 138.74 cM (LG17) with an average length of 110.87 cM (Supplementary Table S1). As for the male map, it contained 4,209 SNP markers and spanned 2830.52 cM with an average marker interval of 0.67 cM (Supplementary Fig. S2). The size of each LG ranged from 45.9 cM (LG14) to 144.83 cM (LG21) with an average length of 117.94 cM (Supplementary Table S1). The female/male length ratio was 0.94, and the female/male marker interval ratio was 0.91.

**Figure 1:**
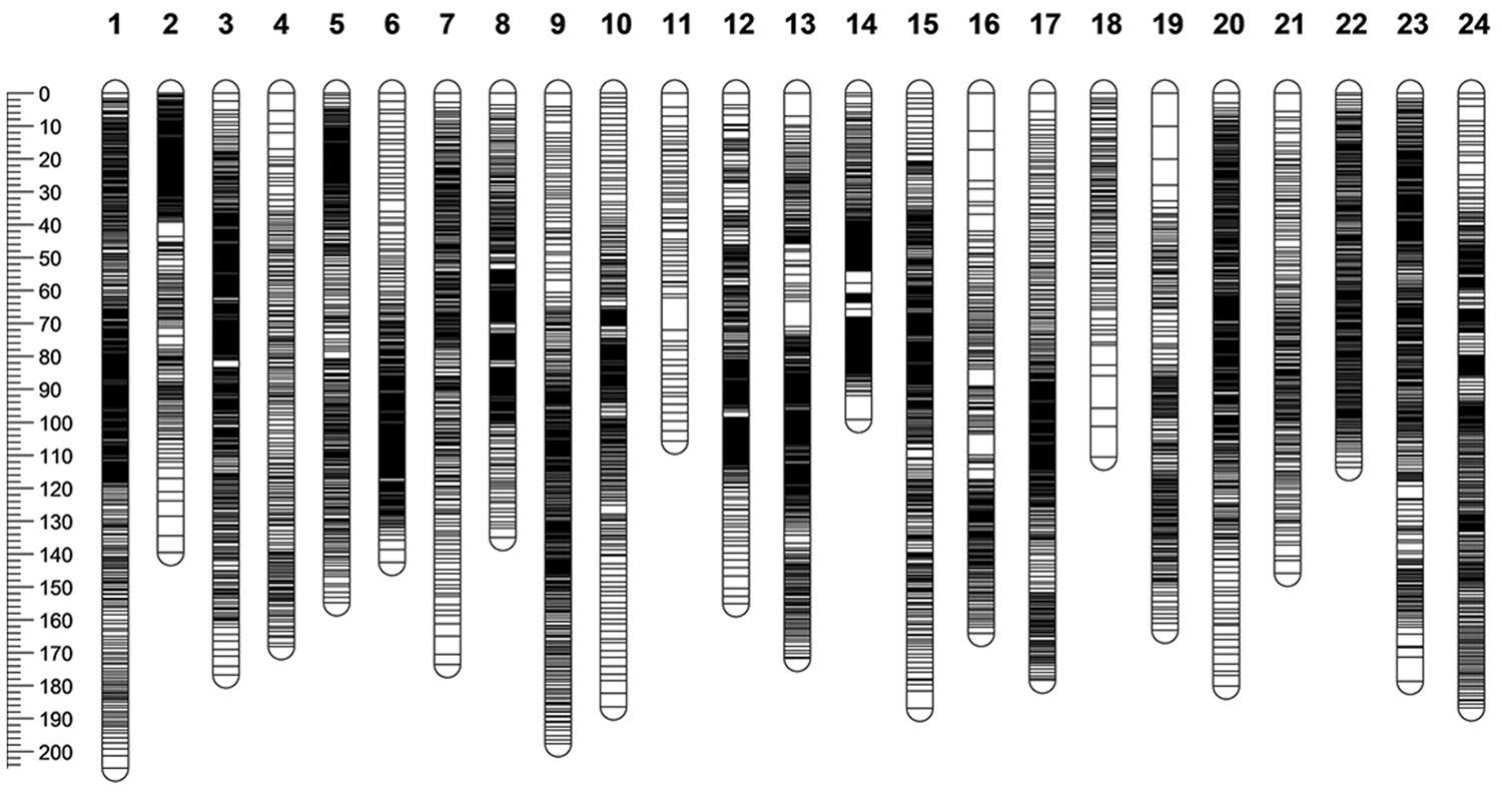
Genetic length and marker distribution of 24 linkage groups in the consensus linkage map of yellow drum. A black bar represents a SNP marker. The scaleplate on the left represents genetic distance (centiMorgan as unit).

Utilizing 533 anchored SNP markers, Sex-specific maps were integrated into a consensus map (Fig. 1). Ultimately, the consensus map consisted of 8,094 SNPs and spanned 3818.24 cM with an average marker interval of 0.47 cM. The shortest LG is 99.08 cM (LG14), and the longest LG is 205.14 cM (LG1) (Table 1). The distribution of all SNP markers on 24 LGs was investigated, which suggested relatively even distribution of markers on the consensus map (Supplementary Fig. S3). Detailed information of all the linkage maps was listed in Supplementary Table S2.

**Table 1:** Details of the consensus map of yellow drum.

LG	Consensus map					
	Map length (cM)	Female-specific markers	Male-specific markers	Shared markers	No. of markers	Marker intervals (cM)
1	205.14	245	258	22	525	0.39
2	139.5	64	196	20	280	0.50
3	176.66	320	164	31	515	0.34
4	168.06	77	114	27	218	0.77
5	154.77	134	240	20	394	0.39
6	142.52	227	89	13	329	0.43
7	173.59	165	149	20	334	0.52
8	134.9	78	251	23	352	0.38
9	197.58	203	186	23	412	0.48
10	186.39	223	63	22	308	0.61
11	105.56	0	54	6	60	1.76
12	155.09	106	164	41	311	0.50
13	171.71	166	213	19	398	0.43
14	99.08	67	301	18	386	0.26
15	186.85	212	196	46	454	0.41
16	164.07	88	103	24	215	0.76
17	178.33	205	189	15	409	0.44
18	110.5	0	78	22	100	1.11
19	163.05	166	62	18	246	0.66
20	180.1	259	154	25	438	0.41
21	145.76	74	131	8	213	0.68
22	113.65	264	38	22	324	0.35
23	178.66	280	146	23	449	0.40
24	186.72	203	196	25	424	0.44
total	3818.24	3826	3735	533	8094	0.47

LG represents linkage group, and cM represents centiMorgan.

Using two different algorithms introduced by Chakravarti *et al*.^48^, genome length was estimated to be 3840.80 cM (G_1_) and 3845.23 cM (G_2_), with an average length of 3843.02 cM as the expected genome length. In summary, the map coverage of our consensus map is 99.4% based on the expected map length, suggesting our linkage map is close to complete.

### Recombination rates between sexes

Comparisons between the two maps uncovered the significant differences in recombination rates and distribution between the two sexes. In all linkage groups between the two sex-specific maps, the average female to male map length ratio is 0.94 (Supplementary Table S1). The LG2, LG7, LG11, LG13, LG19, LG21 and LG24 had more recombination in male than in female, whereas LG6, LG14, LG17 and LG22 showed much higher recombination rate in female (Supplementary Table S1). The distributions of recombination events tended to concentrate towards the end of several LGs, such as LG1, LG4, LG6, LG13 and LG16 in female, and LG1, LG2, LG3, LG5, LG7, LG9, LG10, LG15, LG18, LG20 and LG23 in male (Supplementary Fig. S4). LG2, LG3, LG10, LG12, LG15, LG19, LG21 and LG24 in female-specific map, and LG6, LG8, LG14 and LG22 in male-specific map showed relatively frequent recombination in the middle of the linkage groups (Supplementary Fig. S4). For several LGs, such as LG1, LG3, LG7, LG12, LG13 and LG14, almost no difference was observed in the distribution patterns of recombination events in both sexes (Supplementary Fig. S4).

### Genome assembly and comparative genomics

This linkage map contained 8,094 SNPs, which provided a superb chromosomal framework for genome sequences assembly. As a result, 7,776 SNPs were mapped onto the draft genome and 1,457 scaffolds with total size of 435.2 Mb were placed onto this linkage map, accounting for 80.7% of the genome assembly. The average chromosome length was 18.13 Mb, containing 60.71 scaffolds. The largest chromosome is LG3 (28.55 Mb), containing 504 SNPs anchored to 86 scaffolds. The smallest chromosome is LG24 (3.41 Mb) containing 55 SNPs anchored to 14 scaffolds (Supplementary Table S3).

**Figure 2:**
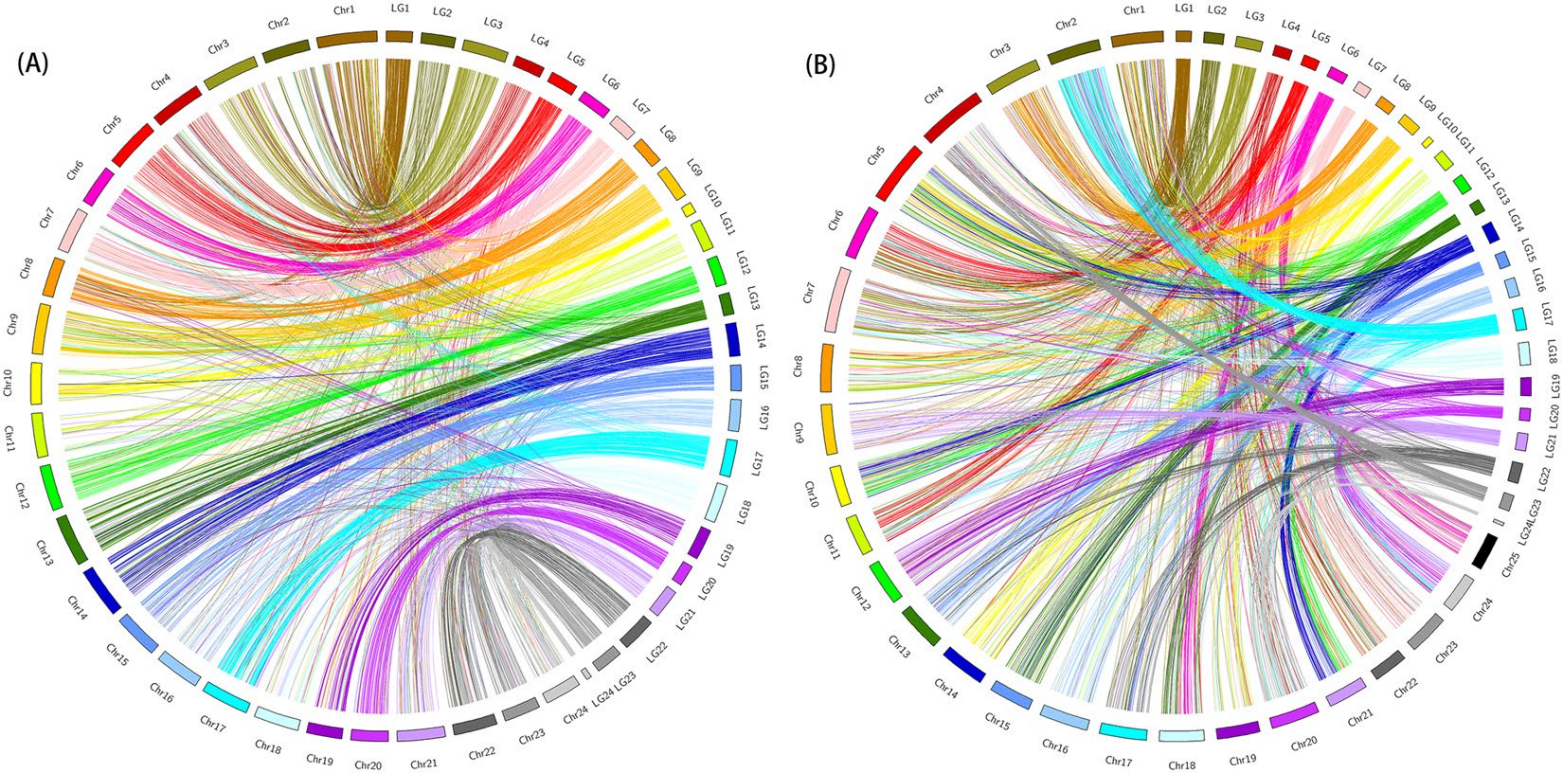
Genomic synteny as shown by Circos diagram for each pair of alignments between yellow drum (right semi-circle) and (**A**) medaka (left semi-circle) and (**B**) zebrafish (left semi-circle).

Likewise, this linkage map can also serve as the chromosomal frame of the yellow drum for comparative analysis of genomic synteny with model fish genomes such as medaka and zebrafish. By in-house script, genome sequences surrounding the SNPs were extracted and aligned to protein sequences of medaka and zebrafish using BLASTx, respectively. As a result, 2,748 1:1 best-aligned orthologues between yellow drum and medaka were observed. Among these orthologues, 2,635 were aligned onto medaka 24 chromosomes and 113 were left on scaffolds. As showed in Fig. 2A, the Circos atlas presented a superb 1:1 inter-chromosomal orthologous pairs between the two genomes. Of the 2,635 orthologous pairs, 1,912 pairs (72.6%) mapped onto those paired chromosomes between yellow drum and medaka, demonstrating superb chromosome-scale synteny. Similarly, comparative analysis between yellow drum and zebrafish genomes identified 2,773 1:1 best-aligned orthologues. Among these orthologues, 2,719 were distributed on zebrafish 25 chromosomes and only 54 were left on scaffolds. As presented in Fig. 2B, more complex genome rearrangements were present between yellow drum and zebrafish than yellow drum and medaka. Although many yellow drum chromosomes tended to be represented on just one paired zebrafish chromosome, we also identified 2:1 chromosomal relationship between yellow drum and zebrafish, such as LG5 vs. Chr6/11 and LG6 vs. Chr18/25. Furthermore, yellow drum genes in LG 12 and 14 had orthologues that broadly distributed among three zebrafish chromosomes (Chr5, Chr10 and Chr21). Unlike the synteny between yellow drum and medaka, we could not identify collinearity in the chromosome scale between yellow drum and zebrafish, suggesting the existence of complicated chromosomal rearrangements post the divergence of zebrafish and yellow drum.

### QTL mapping and association analyses of growth-related traits

**Figure 3:**
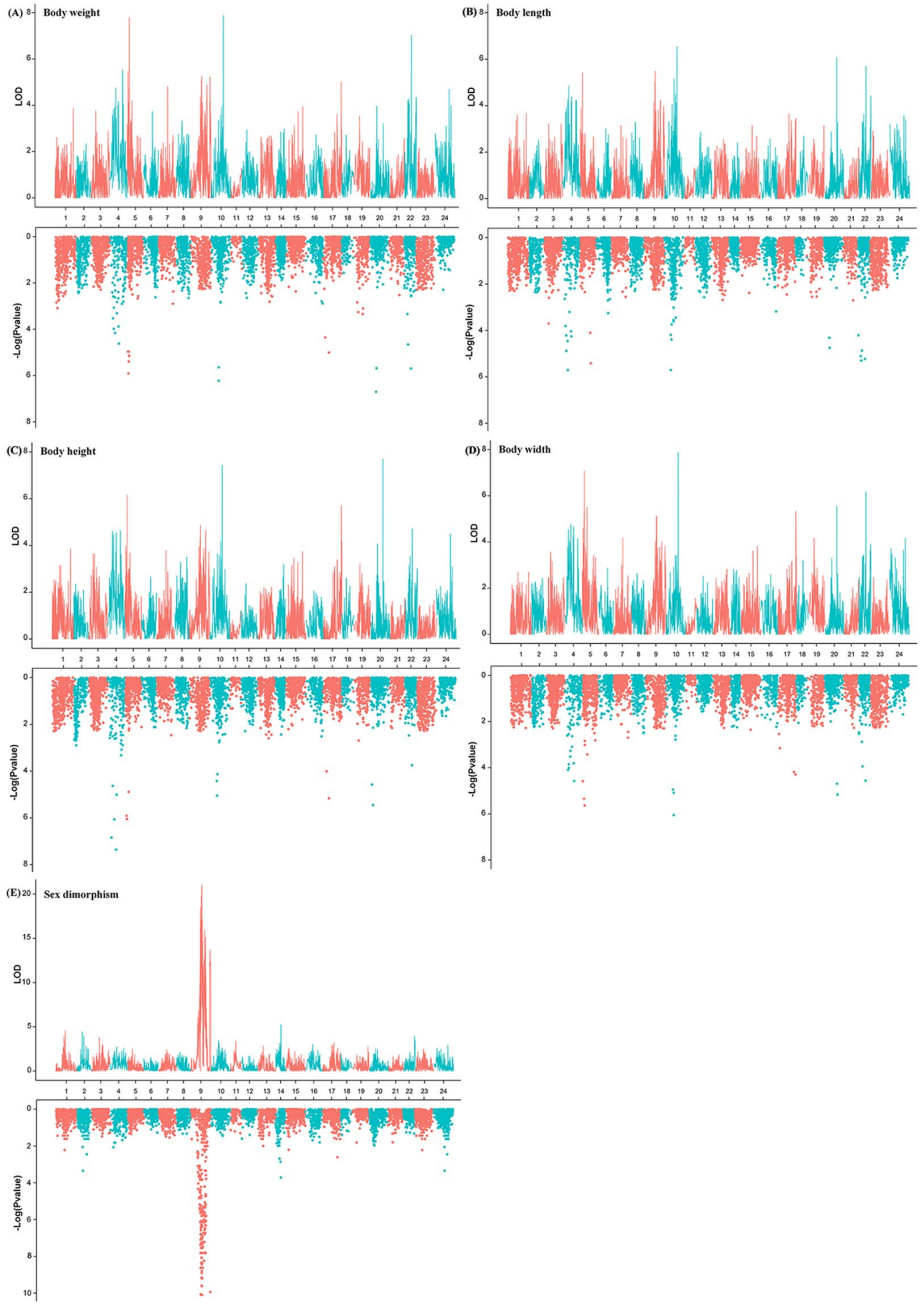
QTL mapping (in the upper portion) and association analyses (in the lower portion) of four growth traits and sex for yellow drum. Significant regions were presented for (**A**) body weight, (**B**) body length, (**C**) body height, (**D**) body width and (**E**) sex dimorphism, respectively. The significance of each locus is represented by the negative logarithms of the estimated p-value for association analyses and LOD value for linkage analyses.

Pairwise comparisons among four growth traits (body weight (BW), body length (BL), body height (BH) and body width (BWD)) using Pearson’s correlation revealed the relatively high correlation among those growth-related traits (Table 2). In general, QTL mapping of those growth traits exhibited quite similar results (Fig. 3A–D and Table 3). As a complementary approach, association analysis revealed a similar distribution pattern across all linkage groups to the results of QTL mapping (Fig. 3). Both the QTL mapping and the association analyses suggested that these growth traits might be regulated by the same set of genes, consistent with the significantly phenotypic correlations among these traits.

**Table 2:** Correlation analyses of four growth traits of yellow drum.

Correlation coefficient	BW	BL	BH	BWD
BW	1			
BL	0.92 (p < 3.77e-46)	1		
BH	0.91 (p < 1.76e-43)	0.84 (p < 1.03e-30)	1	
BWD	0.92 (p < 3.77e-46)	0.84 (p < 1.03e-30)	0.83 (p < 2.11e-29)	1

BW, BL, BH and BWD represent body weight, length, height and width, respectively.

**Table 3:** Summary statistics of the significant QTL for growth and sex in yellow drum.

Traits	QTL name	LG	CI (cM)	NO. of SNPs	LOD	Chromosome-level threshold	Genome-wide threshold	PVE (%)
BW	qBW4a	4	36.27–36.37	0	3.83	3.8	11.6	14.7
	qBW4b		47.32–47.90	2	3.88	3.8	11.6	14.9
	qBW4c		48.87–49.53	1	3.85	3.8	11.6	14.8
	qBW4d		58.36–58.72	1	4.75	3.8	11.6	17.9
	qBW4e		80.36–80.46	1	3.99	3.8	11.6	15.3
	qBW4f		85.3–85.8	1	4.16	3.8	11.6	15.8
	qBW4g		123.6–125.64	3	5.53	3.8	11.6	20.5
	qBW4h		131.21–131.6	2	4.03	3.8	11.6	15.4
	qBW5a	5	7.68–8.11	2	5.47	4.3	11.6	20.3
	qBW5b		17.23–17.41	1	4.6	4.3	11.6	17.4
	qBW5c		19.84–20.22	4	7.78	4.3	11.6	27.6
	qBW10	10	114.36–114.8	1	8.26	6.0	11.6	29.0
	qBW17	17	171.54–171.6	1	5.0	4.9	11.6	18.7
	qBW20	20	112.43–112.73	0	10.65	5.0	11.6	35.7
	qBW22	22	64.60–64.74	1	7.03	6.2	11.6	25.3
BL	qBL4a	4	47.32–47.51	1	4.04	3.9	7.0	15.4
	qBL4b		48.57–49.83	2	4.26	3.9	7.0	16.2
	qBL4c		58.36–58.72	1	4.86	3.9	7.0	18.3
	qBL4d		80.36–80.56	1	4.17	3.9	7.0	15.9
	qBL4e		85.3–86.22	2	4.38	3.9	7.0	16.3
	qBL4f		124.39–125.36	2	4.24	3.9	7.0	16.1
	qBL4g		131.21–131.8	2	4.23	3.9	7.0	16.1
	qBL5	5	20.08–20.22	2	5.41	4.6	7.0	20.1
	qBL10a	10	87.5	1	5.15	5.0	7.0	19.2
	qBL10b		114.36–114.8	1	6.55	5.0	7.0	23.8
	qBL20	20	112.43–112.73	0	6.07	4.5	7.0	22.3
	qBL22	22	64.64–64.74	2	5.7	5.4	7.0	21.0
BH	qBH4a	4	47.22–50.13	5	4.61	3.7	8.3	17.4
	qBH4b		58.36–58.82	1	4.46	3.7	8.3	16.9
	qBH4c		80.36–80.46	1	3.74	3.7	8.3	14.4
	qBH4d		85.30–85.90	1	4.54	3.7	8.3	17.2
	qBH4e		123.89–125.4	2	4.62	3.7	8.3	17.5
	qBH5	5	20.08–20.22	2	6.15	4.3	8.3	22.5
	qBH10	10	114.36–105.0	1	7.3	4.6	8.3	26.1
	qBH17a	17	171.41–171.74	1	5.46	4.7	8.3	20.9
	qBH17b		171.96–172.53	1	5.7	4.7	8.3	21.1
	qBH20	20	112.43–112.73	0	7.48	4.7	8.3	26.7
BWD	qBWD4a	4	34.83–36.67	1	4.54	4.0	6.5	17.2
	qBWD4b		47.22–47.51	1	4.06	4.0	6.5	15.5
	qBWD4c		58.36–58.72	1	4.77	4.0	6.5	18.0
	qBWD4d		80.46–80.56	1	4.22	4.0	6.5	16.1
	qBWD4e		85.3–86.42	2	4.66	4.0	6.5	17.6
	qBWD4f		124.19–124.69	0	4.14	4.0	6.5	15.8
	qBWD5a	5	7.68–8.01	2	4.59	4.1	6.5	17.3
	qBWD5b		17.23–17.41	1	5.25	4.1	6.5	19.6
	qBWD5c		19.74–20.32	4	7.08	4.1	6.5	25.4
	qBWD5d		45.85–46.25	2	5.49	4.1	6.5	20.4
	qBWD10	10	114.36–114.93	1	7.88	5.1	6.5	27.9
	qBWD17	17	171.54–171.74	1	5.32	4.7	6.5	19.8
	qBWD20	20	112.43–112.73	0	5.54	4.8	6.5	20.5
	qBWD22	22	64.6–64.74	1	6.17	5.2	6.5	22.6
SD	qSD9a	9	67.69–68.89	2	5.27	4.6	6.8	19.6
	qSD9b		69.06–69.83	2	6.06	4.6	6.8	22.2
	qSD9c		71.02–71.84	1	5.31	4.6	6.8	19.8
	qSD9d		74.97–78.63	8	6.46	4.6	6.8	23.5
	qSD9e		78.82–81.29	3	6.05	4.6	6.8	22.2
	qSD9f		85.34–86.19	2	8.43	4.6	6.8	29.5
	qSD9g		86.39–95.84	28	18.48	4.6	6.8	53.5
	qSD9h		96.28–117.31	95	20.99	4.6	6.8	58.1
	qSD9i		118.75–123.72	8	11.98	4.6	6.8	39.2
	qSD9j		125.24–145.7	89	15.36	4.6	6.8	47.1
	qSD9k		145.99–148.36	3	10.62	4.6	6.8	35.6
	qSD9l		149.99–153.58	4	7.16	4.6	6.8	25.7
	qSD9m		183.59–192.41	2	13.66	4.6	6.8	43.3

BW, BL, BH, BWD and SD represent body weight, length, height and width and sex dimorphism, respectively. LD: linkage group; CI: confidence interval; LOD: logarithm of odds; PVE: phenotypic variance explained.

In total, 22 QTLs associated with growth surpassed chromosome-wide significance. More specifically, 6 QTL regions including 21 SNPs were identified for body weight, distributing on 6 LGs including LG4, LG5, LG10, LG17, LG20 and LG22 (Fig. 3A and Table 3). The most significant QTL qBW20e located on LG20 at 112.43–112.73 cM presented the highest LOD score of 10.65, explaining 35.7% of phenotypic variations. For body length, 5 QTLs were identified on 5 LGs (LG4, LG5, LG10, LG20 and LG22) harboring 17 significant SNPs (Fig. 3B and Table 3). The most significant QTL qBW10c, with a highest LOD value of 6.55, located on LG10 at 114.36–114.80 cM, explained 23.8% of phenotypic variations. For body height, 15 SNPs from 10 QTLs were identified on 5 LGs (LG4, LG5, LG10, LG17 and LG20) (Fig. 3C and Table 3), and the most significant QTL qBH20 (perfectly overlapped with qBW20) explained 26.7% of the total phenotypic variations. For body width, we identified 6 QTLs encompassing 18 SNPs on 6 LGs (LG4, LG5, LG10, LG17, LG20 and LG22) (Fig. 3D and Table 3). The QTL qBWD10c located on LG10 at 114.36–114.93 cM, with the highest LOD value of 7.88, explained 27.9% of the total phenotypic variations. Of those QTLs for the four growth traits, significant portions that either shared or overlapped among the four traits were observed, suggesting that those traits might be regulated by the same set of genes in the yellow drum.

Knowledge of the functional genes in other species during development and growth would be helpful for seeking the growth-related genes in yellow drum. A total of 10, 9, 9 and 9 genes were identified for BW, BL, BH and BWD through screening the QTL intervals, respectively (Supplementary Table S4). Interestingly, most of these genes were shared among the four growth traits. We identified the *PLA2G4A* (phospholipase A2 group IVA) gene from QTL qBW4b, which also located in the confidence intervals of qBL4a, qBH4a and qBWD4b. Functional analysis of *PLA2G4A* showed that it is involved in development and growth-related biological processes, including brown fat cell differentiation (GO: 0050873) and regulation of cell proliferation (GO: 0042127) (Supplementary Table S4). It is thought that cytosolic group IVA phospholipase A2 is the pivotal enzyme in receptor-mediated arachidonic acid (AA) mobilization and attendant eicosanoid production and plays a critical role in early adipocyte differentiation and obesity^49^. We thus speculated that *PLA2G4A* might play a similar function in regulating the development and growth in the yellow drum.

We also identified the *BRINP3* (BMP/retinoic acid inducible neural specific 3) gene which located in the confidence intervals of qBW4g, qBL4f and qBH4e. The Gene Ontology of *BRINP3* indicated that it participated widely in growth, including cell cycle (GO: 0007049) and negative regulation of mitotic cell cycle (GO: 0045930) (Supplementary Table S4). It was reported that BRINPs exerted a similar proliferation inhibition function in non-neural tissues, despite that the predominant expression of *BRINP* family genes in the nervous system^50^. Therefore, we suspected that *BRINP3* is related to growth of the yellow drum. Furthermore, we identified *P2RY1* (purinergic receptor P2Y1) from QTL qBW5a, which overlapped with QTL qBWD5a. The Gene Ontology assigned *P2RY1* to several biological processes associated with growth, including eating behavior (GO: 0042755) and response to growth factor (GO: 0070848) (Supplementary Table S4). Previous findings demonstrated that *P2RY1* broadcasts mitogenic signals and facilitate mitosis^51^. Hence, we suggested this gene was associated with growth of the yellow drum.

Furthermore, *ANO8* (anoctamin 8) (located in qBW4f, qBL4e, qBH4d and qBWD4e) and *ZCCHC11* (zinc finger CCHC-type containing 11) (located in qBL4g) might also be functional in growth in the yellow drum, based their functions in other species. *ANO8* encodes TMEM16 family protein, which was thought to be highly expressed during murine embryogenesis^52^. The Zcchc11 enzyme is implicated in microRNA regulation, and it was suggested to mediate cytokine and growth factor expression^53^.

### Mapping sex-controlling locus

The sex determination mechanism and gene(s) of the yellow drum is still unknown. In this study, we identified significant QTLs (chromosome-wide p < 0.05) encompassing 247 SNPs for sex dimorphism on LG9. The most significant QTL qSD9h located on LG9 at 96.28–117.31 cM with the highest LOD score of 20.99, explaining more than half (58.1%) of the total phenotypic variations. Association analysis between SNP genotypes and sex dimorphism demonstrated high concordance in QTL localization between the two approaches (Fig. 3E and Table 3).

We screened the QTL intervals and identified 124 candidate genes underlying sex dimorphism. We found the *DMRT* gene family, including *DMRT1*, *DMRT2* and *DMRT3*, from the most significant QTL qSD9h. The Gene Ontology analysis directly assigned those genes to the biological processes of sex differentiation (GO: 0007548) (Supplementary Table S4). It was reported that the *DMRT1* gene is found in a cluster with two other members of the gene family, sharing a zinc finger-like DNA-binding motif (DM domain). The DM domain-containing genes are ancient, conserved components of the vertebrate sex-determining pathway is numerous species such as flies and nematodes^54^. In addition, *DMRT* genes exhibit a gonad-specific and sexually dimorphic expression pattern in many animals^55–60^. Therefore, we speculated that those *DMRT* genes might potentially associate with sex dimorphism in yellow drum as well.

## Discussion

Genetic maps are important genomic resources for genomic and genetic studies, which were widely used for genome assembly, functional gene mapping and comparative genome analysis. In the present work, we reported the construction of a high-density linkage map for yellow drum, containing 8,094 SNP markers with an overall genetic length of 3818.24 cM and an average marker interval of 0.47 cM. As far as we know, this is the first high-density genetic linkage map for yellow drum, and it will fill the gaps in linkage map construction and gene mapping of yellow drum. With this linkage map, QTL mapping, positioning of candidate genes, marker-assisted selection and comparative analysis of genomic synteny can be performed and the assembly of chromosome-scale reference genome can be achieved in the yellow drum.

To obtain sufficient genetic segregation loci for linkage analysis, F2, backcross (BC) families, recombinant inbred lines (RIL) and double haploid (DH) were commonly used for linkage map construction^10^. Actually, it is an extremely time-consuming work to construct RIL or DH family in teleost due to long generation interval. In this study, F1 family was used to construct the linkage map employing double pseudo-testcross strategy, which was first proposed by Grattapaglia and Sederoff ^61^ and have been widely applied to the construction of linkage maps in many aquaculture species^7,10,62^. Whole genome resequencing was performed for all fish from the mapping family, providing abundant SNPs for linkage mapping. In order to get a high-quality linkage map, we applied high stringency to the data filtration (SNP calling rate >95% and MAF >5%) and finally retained about 1.2 million SNP markers, in which we can sufficiently select sites of testcross pattern in a 1:1 ratio between two parents. Sex-specific maps were firstly constructed with these testcross loci. Subsequently, the two sex-specific maps were integrated into a consensus map through a set of shared markers. Recombination rate comparison indicated that both sex-specific maps and the consensus map of yellow drum were highly consistent with each other, suggesting the high quality of the consensus map.

In this study, the female map was slightly shorter than the male map with a female to male (F/M) length ratio of 0.94 (2660.82 cM vs 2830.52 cM). This result was inconsistent with the pattern between sexes in several reported fish (zebrafish: 2.7 F/M ratio^63^; Atlantic salmon^11^: 8.3 F/M ratio^64^) and most studied mammals (human: 1.6 F/M ratio^65,66^; mouse: 1.3 F/M ratio^67^; dog: 1.4 F/M ratio^68^). However, our observation is consistent with several previous reports. The male recombination rate was found to be larger than that of female in sheep^69,70^ (0.77 F/M ratio) and cattle (0.92 F/M ratio^71^; 0.91 F/M ratio^72^). Also, the longer male genetic map was also observed in common carp (0.94 F/M ratio^10^). The differences in recombination rate between sexes have been found to correlate with the length of the synaptonemal complex^67,73^. Previous karyotype analysis showed that yellow drum lacks obvious heteromorphic sex chromosomes^47^, indicating the tiny genetic difference in sex chromosome of yellow drum, which might be an explanation for the longer male map in our study contradicts Haldane’s prediction of a higher recombination rate in the homogametic versus heterogametic sex^74^.

The high-density linkage map served as a chromosome framework and the draft genome sequences of the yellow drum were anchored onto 24 linkage groups. Ultimately, 1,457 scaffolds with total length of 435.15 Mb were anchored onto the genetic map, accounting for 80.7% of the genome assembly. In this study, abundant SNPs were used for anchoring the scaffolds. However, the assembly rate was relatively low. Such a phenomenon might result from the following two reasons. Firstly, the current assembled genome sequence of yellow drum is much fragmented. The contig N50 length is 50.3 kb (unpublished data) in the draft assembly of the yellow drum. We expected a new version of the reference genome employing optimized assembly algorithms to be completed soon, providing a possibility to anchor more genome sequences to chromosomes. Secondly, although over 8,000 SNP markers were used to construct this linkage map, these markers were not evenly distributed throughout the entire genome. For example, almost 10 cM gaps were observed in LG16 and LG19.

The generation of a high-density linkage map allowed us to perform QTL mapping of the growth traits of the yellow drum. Growth traits are of great interest to breeders due to their high commercial significance in aquaculture. QTL mapping stands for an efficient approach to the identification of genetic loci underlying these traits^75^. In this study, 22 growth-related QTLs were detected in the linkage groups LG4, LG5, LG10, LG17, LG20 and LG22 and confirmed by the association analysis. Gene Ontology analysis provides hints that *PLA2G4A*, *BRINP3* and *P2RY1* in these QTLs participate in growth, which suggest that these genes might constitute valuable resource for further marker-assisted selection in breeding programs of yellow drum and further fine-mapping is needed to identify the causative gene(s)/variant(s). Furthermore, we also found that most of these QTLs were overlapped among the four growth traits. One reason for this result may be the relatively high phenotypic correlation among the four traits in the yellow drum. Therefore, we suggest that QTL in one trait may be responsible for other three, and selection for one of the four traits may result in improvement of other growth traits in the breeding of the yellow drum. Another reason may be that growth-related traits in the yellow drum were controlled by only a few major genes in the genome. This pattern was similar to that of QTL for growth-related traits in bighead carp^9^.

Recent study indicated that yellow drum adopt the male heterogametic XX/XY sex determination system^76^. However, the genes and mechanisms underlying sex differentiation of the yellow drum are still largely unknown. Yellow drum exhibits a sex-dependent dimorphic growth pattern where females grow much faster than males^76^. An understanding of sex determination mechanisms in the yellow drum is crucial to improve the growth traits related with sex dimorphism and set up all-female or all-male production system in the fisheries. In this study, QTLs for sex dimorphism were mapped on LG9, and a series of candidate genes including *DMRT1*, *DMRT2* and *DMRT3* were identified in these QTL regions. The *DMRT* genes exhibit a gonad-specific and sexually dimorphic expression pattern, and work as sex determination genes in several species, such as medaka^55^, zebrafish^59^, mouse^57^ and chicken^57^. We suspect that the *DMRT* genes might play important roles in sex determination in the yellow drum and further fine mapping is needed to identify the causative gene(s)/variant(s). We expect that these potential causative genes will lay a foundation for understanding the sex determination mechanism of the yellow drum.

In conclusion, we reported the construction of a high-density genetic linkage map for the yellow drum. This map contained 8,094 SNPs and spanned 3818.24 cM with an average marker interval of 0.47 cM. With this map, we anchored 80.7% of the genome scaffolds onto 24 chromosomes. Comparative genomic analyses were performed between yellow drum and two model fish species, i.e., medaka and zebrafish, demonstrating superb chromosome-scale synteny between yellow drum and medaka. Finally, we mapped the QTL loci and identified candidate genes for growth and sex. As we demonstrated here, this linkage map can serve as an important tool for QTL mapping of economically important traits as well as for genome assembly. The candidate genes and markers for growth and sex will facilitate the ongoing genetic breeding programs of the yellow drum.

## Methods

### Ethics Statement

The study and all experimental protocols were approved by the Animal Care and Use Committee of the Fisheries College of Jimei University. The methods were performed in accordance with relevant guidelines.

### Mapping family and genome resequencing

A F1 full-sib yellow drum family including 2 parents and their offspring was constructed in an experimental breeding farm in Ningde, Fujian province, P.R. China. All the fish were fed with standard feed. 111 offspring were randomly selected 18 months post hatch from the mapping family for subsequent genotyping and phenotype measure. The body weight was measured on the electronic scales. The body length, body height and body width were measured using the vernier caliper. Sex of each individual was determined through examining the gonads by dissection. Genomic DNA was isolated from dorsal fins of 2 parents and 111 offspring using TIANamp Genomic DNA Kit (TIANGEN, Beijing, China). After quantification by Nanodrop-1000 spectrophotometer (Thermo Scientific, Wilmington, DE, USA) and integrity examination by agarose gel electrophoresis, DNA samples were diluted and submitted for sequencing. All samples were sequenced on Illumina HiSeq X10 platform with 150-bp pair-end sequencing mode.

### SNP calling

All filtered high-quality reads of 113 samples were aligned to the draft genome of the yellow drum using BWA v0.7.17^77^. The aligned bam files were sorted by coordinates using SAMTOOLS v1.4^78^. The Genome Analysis Tool Kit (GATK) package v3.6.0^46^ was used to further process bam files and SNP calling. In detail, duplicated reads were removed, reads around insertions/deletions were realigned, and base quality were recalibrated using default parameters of GATK. SNPs were firstly called using SAMTOOLS with mpileup flag to provide a reference VCF for base quality recalibration. All variants were finally generated using HaplotypeCaller method in GATK with emitting and calling standard confidence thresholds at 10.0 and 30.0, respectively. The SNP loci of depth less than 20 and quality score less than 60 were removed from the final list to obtain high accuracy markers for subsequent analysis.

### Linkage mapping

Firstly, SNPs were grouped into three categories based on their segregation patterns: AB × AA or AB × BB (heterozygous only in female parent, 1:1 segregation in the offspring), AA × AB or BB × AB (heterozygous only in male parent, 1:1 segregation in the offspring), and AB × AB (1:2:1 segregation in the offspring). Both female and male linkage maps were constructed using JoinMap 4.0 software. A logarithm of odds (LOD) threshold of 8.0 was selected to assign markers into 24 linkage groups. Recombination rates were calculated by the regression mapping algorithm, and map distances were converted by Kosambi mapping function. A consensus map was established by integrating the sex-specific maps through shared markers using the MergeMap software (http://www.mergemap.org/). All genetic linkage maps were drawn using MapChart 2.2^79^ software.

### Characterization of recombination between sexes

Different markers were used when constructing the sex-specific genetic maps and thus the recombination rates could not be directly compared using marker intervals. We investigated the difference in recombination rates between sexes employing the method proposed by Gonen^11^. Followed the method described by Wang *et al*.^7^, each linkage group was divided into at least 10 intervals of the same size and the number of intervals between each pair of sex-specific linkage group was the same. The percentage of markers mapped to each interval was calculated and compared across each linkage group pair of sex-specific LGs.

### Assembling scaffolds and comparative genomics

By in-house script, genome sequences surrounding the SNP loci (50 bp on each side) were extracted to represent each SNP in the consensus map. Subsequently, these sequences were mapped to the draft genome of the yellow drum using BLASTN. The scaffolds were assigned to LGs based on their best match (alignment length ≧100 bp and gap length <1 bp). The position and orientation of each scaffold was determined with at least two SNPs.

In order to encompass potential protein coding sequences, genome sequences with a length of 3,001 bp surrounding the SNPs were extracted from the reference genome of yellow drum by in-house script. BLASTx searches were performed using these sequences against protein sequences of medaka and zebrafish with e-value cutoff of e^−10^. The zebrafish and medaka protein sequences were downloaded from Ensembl databases (http://asia.ensembl.org/info/data/ftp/index.html).

### QTL mapping and association analyses of growth-related traits and sex dimorphism

Pairwise correlations among four growth traits were performed across all progenies using Pearson correlations. Assuming that the correlation of two random traits in the mapping family was equal to zero, we tested whether the correlation of two traits was statistically significant by comparison to zero with *Student*’*s t*-test (p-value ≤ 0.05). QTL mapping was performed using MapQTL 6.0 software package with “composite interval mapping” (CIM) and “restricted multiple QTL model (MQM) mapping” algorithms^80^. LOD significance thresholds were calculated by 2,000-permutation tests at a chromosome-wide significance level of α < 0.05. As a complementary approach to QTL mapping, association analysis was performed between genotypes and phenotype using Plink 1.07^81^. The candidate genes in the QTL intervals were identified from the aligned scaffolds that were annotated with multiple tissues pooled RNAseq data^82^. GO annotation was performed with NCBI2R (http://ncbi2r.wordpress.com/) as implemented in the R package^83^.

## Electronic supplementary material

Supplementary Information

Dataset 1

Dataset 2

## Data Availability

The raw sequencing data were archived at the NCBI Sequence Read Archive (SRA) under Accession Number PRJNA431723.
