# Supplementary material for: A high-density genetic linkage map and QTL mapping for growth and sex of yellow drum (Nibea albiflora)
Source: Sci Rep. 2018 Nov 22;8:17271. doi: 10.1038/s41598-018-35583-1 (PMC6250659; doi:10.1038/s41598-018-35583-1)

A high-density genetic linkage map and QTL mapping for growth and sex of yellow drum (*Nibea albiflora*)

Changliang Qiu[1†], Zhaofang Han[1†], Wanbo Li[1], Kun Ye[1], Yangjie Xie[1] & Zhiyong Wang[1, 2 *]

[1]Key Laboratory of Healthy Mariculture for the East China Sea, Ministry of Agriculture; Fisheries College, Jimei University, Yindou Road, Xiamen, Fujian, P.R.China.

[2]Laboratory for Marine Fisheries Science and Food Production Processes, Qingdao National Laboratory for Marine Science and Technology, Qingdao 266235, PR China

[†] These authors contributed to this work equally.

*Corresponding author: Zhiyong Wang, telephone: +86-18950124893, email: zywang@jmu.edu.cn

Supplementary data:

Supplementary Figure S1: the female-specific genetic linkage map of yellow drum.

Supplementary Figure S2: the male-specific genetic linkage map of yellow drum.

Supplementary Figure S3: The patterns of marker distribution on each linkage group.

Supplementary Figure S4: The distribution patterns of recombination events across each linkage group of sex-specific genetic maps of yellow drum.

Supplementary Table S1: Summary of the sex-specific linkage maps of yellow drum.

Supplementary Table S2: Detailed information of the sex-specific and consensus maps of yellow drum.

Supplementary Table S3: Details of physical map of yellow drum anchored to linkage map.

Supplementary Table S4: GO annotation of the SNPs markers in the significant QTL regions.

Figure S1: the female-specific genetic linkage map of yellow drum.

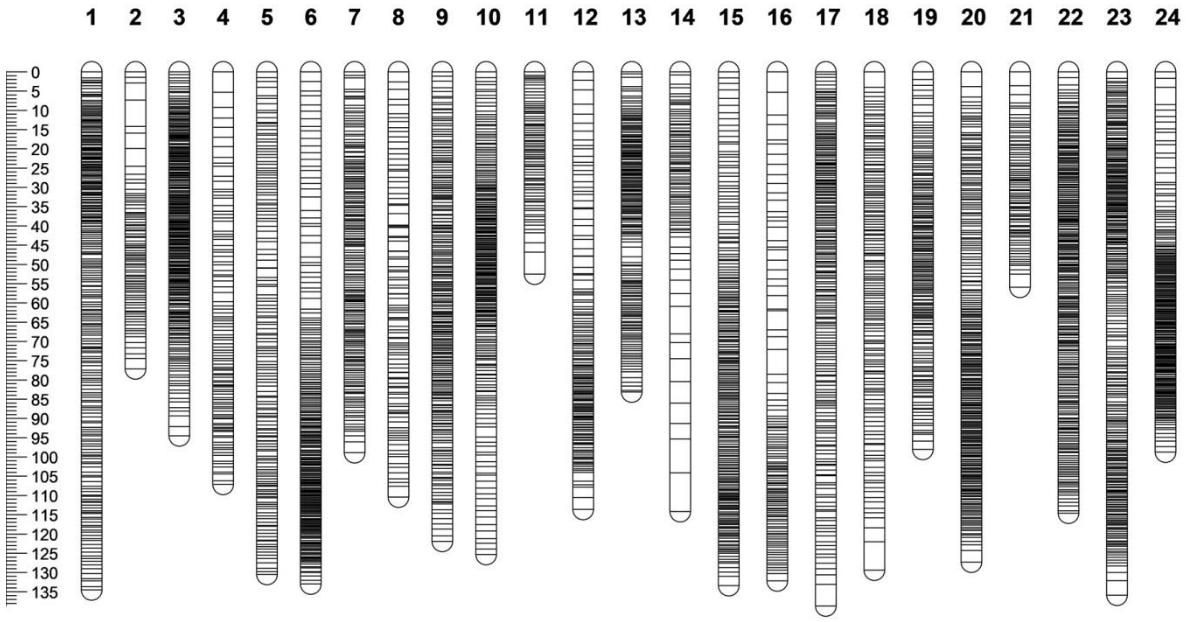

Figure S2: the male-specific genetic linkage map of yellow drum.

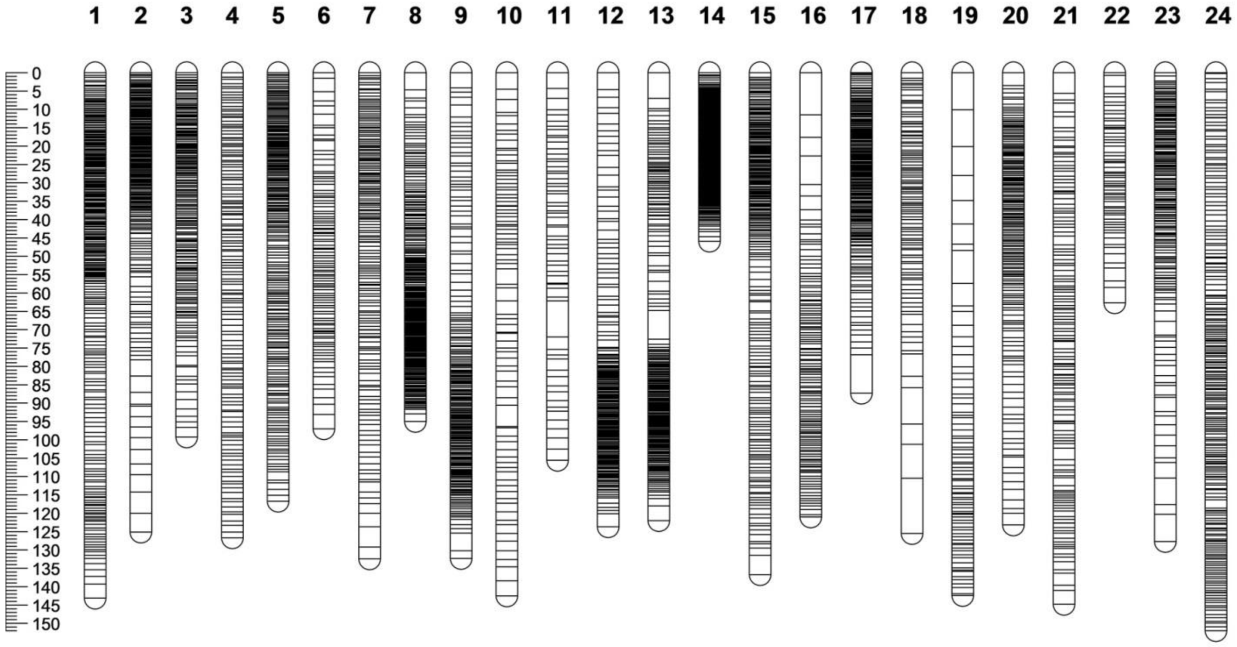

Figure S3: The patterns of marker distribution on each linkage group. The X-axis represents marker orders on each linkage group. The Y-axis represents SNP marker position (cM) on each linkage group.

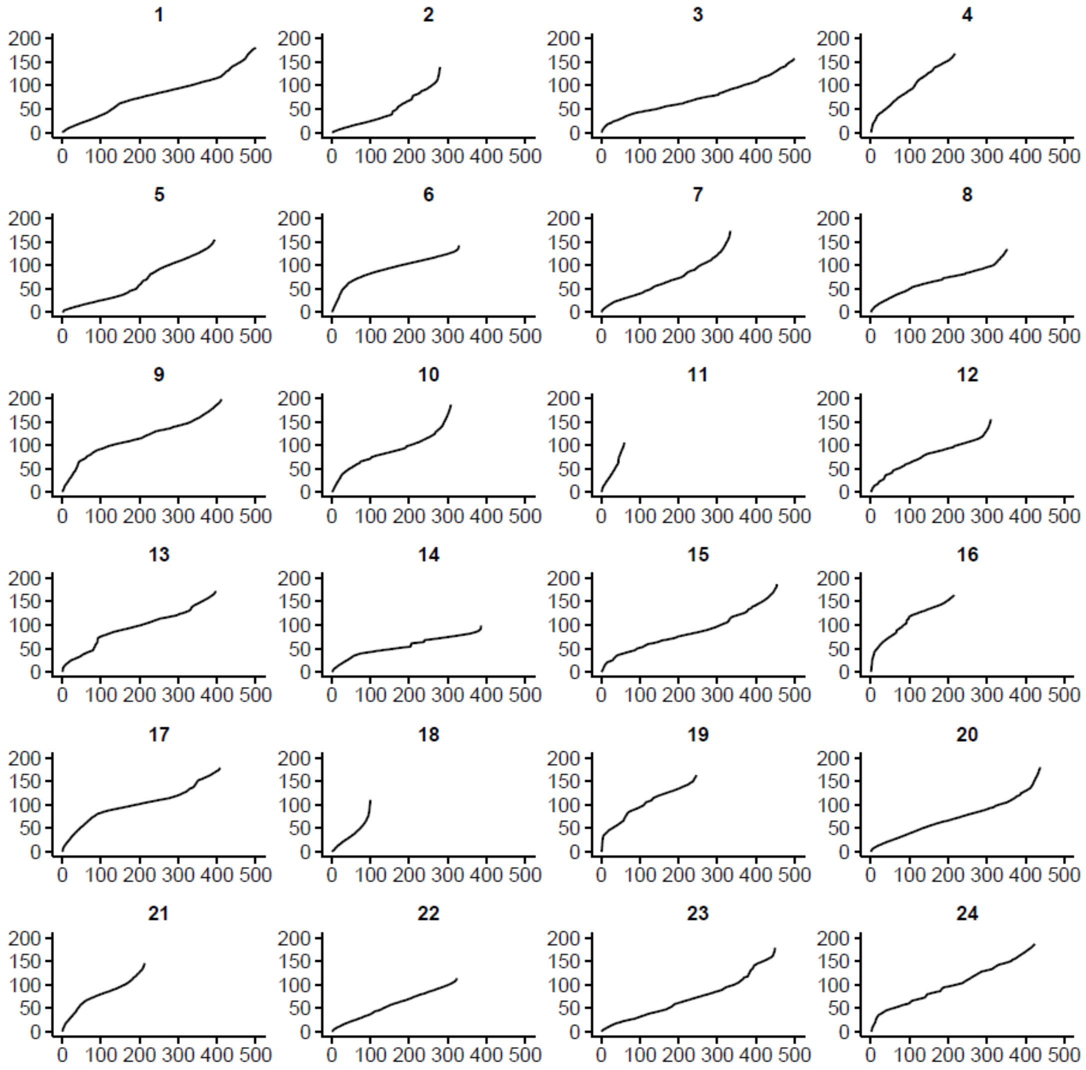

Figure S4: The distribution patterns of recombination events across each linkage group of sex-specific genetic maps of yellow drum.

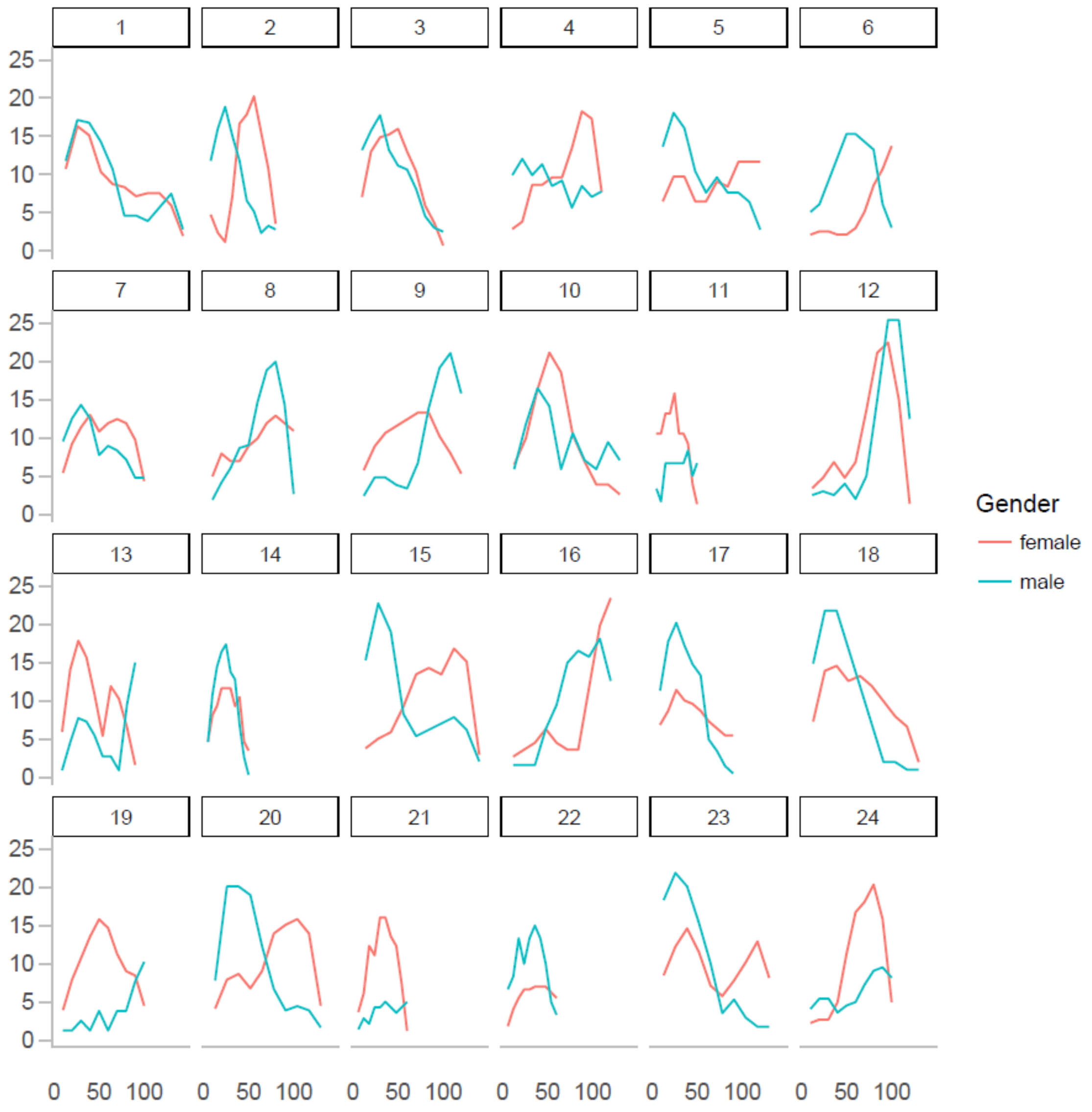

Supplement: Supplementary file 1 — Supplementary Information [file 41598_2018_35583_MOESM1_ESM.pdf]
